# Kinetics and adsorption isotherms studies for the effective removal of Evans blue dye from an aqueous solution utilizing forsterite nanoparticles

**DOI:** 10.1038/s41598-024-73697-x

**Published:** 2024-10-17

**Authors:** Ahmed Magdy, Maysa R. Mostafa, Saied A. Moustafa, Gehad G. Mohamed, Omar A. Fouad

**Affiliations:** 1 Chemistry Department, Faculty of Science, Cairo University, Giza, 12613 Egypt; 2 Nanoscience Department, Basic and Applied Sciences Institute, Egypt-Japan University of Science and Technology, New Borg El Arab, 21934 Alexandria Egypt

**Keywords:** Forsterite nanoparticles, Evans blue dye, XRD, AFM, TEM, BET, Contact angle, Pseudo-second-order reaction, Langmuir model, Nanoscale materials, Analytical chemistry, Environmental chemistry

## Abstract

In the present day, water treatment has emerged as a significant global concern, particularly due to the proliferation of pollution sources. The utilization of dyes, such as Evans blue, in several industries is among the most significant contributors to these pollutants. Forsterite nanoparticles were synthesized by the sol-gel technique and calcined at different temperatures to determine the optimum temperature at which pure nanoforsterite was obtained. Then, it was analyzed using X-ray diffraction (XRD), atomic force microscope (AFM), transmission electron microscope (TEM), Brunauer-Emmett-Teller (BET) , contact angle, and zero-point charge. The adsorption capability of forsterite nanoparticles (Nps) was evaluated by a batch adsorption experimental method to remove Evans blue dye (EBD). Parameters such as agitation speed, dosage of forsterite Nps, pH, and contact time were considered at ambient temperature. At pH = 3, dose of Nps = 1 g/L, and 600 rpm within 10 min, the results indicated a removal rate of around 100%. Furthermore, it was shown that the material may be employed for 3 cycles with a removal rate of 90%. Multiple kinetic and isotherm models, including Langmuir, Temkin, and Freundlich models, were used to analyze the results and clarify the mechanism of the adsorption phenomena. The findings from the isotherm and kinetic studies indicated that the system conforms to Langmuir and pseudo-second-order, respectively.

**Supplementary Information:**

The online version contains supplementary material available at 10.1038/s41598-024-73697-x.

## Introduction

Water is regarded as one of the most significant resources on the planet. It is required in practically every aspect of our lives, including the home, business, and agriculture. Animals, plants, and other humans cannot survive without fresh water. Economic growth is boosted when clean drinking water is readily available. Although water covers about 70% of Earth, only a small portion of it is accessed^1^. The combination of rapid population expansion and increasing living standards, together with several other human causes, is driving a surge in the production and consumption of daily necessities. Consequently, this is leading to the entry of detrimental pollutants into water systems. Research has shown that a significant number of radioactive elements, endocrine-disrupting chemicals, food preservatives, personal care items, dyes, and pharmaceutically active molecules may be found in water and wastewater. Emerging contaminants, which refer to these compounds, provide a significant risk to both persons and the environment^2^.

The United States Geological Survey defines emerging contaminants as substances or microorganisms that are not often seen in the environment but have the potential to seriously affect both human health and the ecosystem, even at extremely low concentrations^3^. Most environmental pollutants are polar and highly soluble, making them common in water and wastewater systems. Even at low concentrations, the presence of certain environmental pollutants can induce chronic harmful effects in people and animals^4^.

Dyes have increased in popularity in recent decades owing to the fast growth of dye-based businesses and increasing demand for garments and textiles. Synthetic dyes have accumulated in significant quantities in the environment. These colors are used in the textile business, as well as in rubber, food, paper, cosmetics, and other industries. Even at low concentrations, the presence of colors in effluents is quite noticeable, and these pigments’ oxidation products are typically carcinogenic. Furthermore, these dyes have a variety of hazardous effects on genotoxic, living organisms, including humans, with numerous dyes exhibiting cytotoxic, neurotoxic, mutagenic, hypersensitivity, and mitochondrial toxicant qualities. To address this concern, several countries have enacted rigorous environmental rules governing the textile sector^5^. One of the most prevalent contaminants found in wastewater discharged from a variety of sectors, including dyeing (21%), tanneries (8%), paper (10%), textiles (54%), and dye manufacture (7%), is organic dye. Around 700,000 tons of commercial dyes are manufactured annually. Most dyes withstand environmental stresses including light, temperature, and chemical agents, and are both soluble and persistent in water. Although the typical dye concentration liberated from textiles is roughly 300 mg/L, they are visible in water at low quantities above 1 mg/L and pose a major risk to the environment and human health. The removal of dyes from water is made more difficult by the coexistence of many dye categories, such as basic, acidic, reactive, and direct dyes. Notably, the basic and diazo direct dyes showed the highest rates of toxicity^6^. Such as Evans blue dye (EBD) which is classified as a hazardous dye that affects lung function, the liver, kidneys, skin, and intestines. It also has long-term health effects. In addition to irritating the skin when applied in large amounts, it is also recognized to be genotoxic and carcinogenic. To prevent wastewater from being dumped into the environment, it is crucial to remove certain colors^7^.

Traditional approaches to water treatment include electrochemical approaches, degradation, chemical reduction^8,9^, membrane filtering^10,11^, anaerobic or aerobic treatment, coagulation, flocculation^10^, and adsorption^12^. The adsorption technique is still the most used method for dealing with water contamination^6^ but these traditional approaches have drawbacks such as large equipment, cost effect, and fast contamination. Thus, the necessity to investigate novel and effective treatment strategies is imperative^13^. Adsorption has been developed and used extensively in the current day to eliminate water contaminants because of its durability, few byproducts, and great efficiency. Targeted pollutants can be trapped by physical force or chemical binding; the adsorption of water contaminants is controlled by the surface area, specific active site, and selectivity of a porous adsorbent^14^.

Ceramic nanomaterials have been successfully used for water treatment. The main constituents of ceramic nanomaterials, which are inorganic systems with porous properties, are the oxides, phosphates, carbides, and carbonates of metals and metalloids like silicon, calcium, titanium, and so on. They have a wide range of uses because of their many favorable properties, including their excellent heat resistance and chemical inertness. Nanoparticles’ size range, surface properties, porosity, surface area to volume ratio, and other characteristics must all be controlled for them to be effective in water treatment. The technique of preparation and precise control over process factors are crucial in attaining these desirable qualities^15,16^. Ceramic materials also provided more active sites for the removal of contaminants from water such as Forsterite.

Forsterites have the chemical composition Mg_2_SiO_4_ and are a crucial constituent of both the olivine and pyroxene mineral families regarded as nanoceramic materials because of their exceptional physical properties^17^. They have high refractoriness (≥ 1890 °C), low dielectric constant, excellent thermal insulation, low thermal expansion coefficient, and good chemical stability. Forsterites are widely used in the production of various technical components, including biomaterials, dielectric substrates, refractory materials, optical devices, pigments, solid oxide fuel cells (SOFC), and composite materials^18,19^. Thus, this work aims to synthesize forsterite nanomaterial by using the sol-gel method and studying its structural, microstructural, surface area, and hydrophilicity for its use as an adsorbent to remove EBD from wastewater. Factors that affect this process were studied and optimized in addition to describing the kinetic and isothermal models that fit the adsorption process.

## Results and discussion

### Characterization of the sorbent

#### XRD

**Fig. 1:**
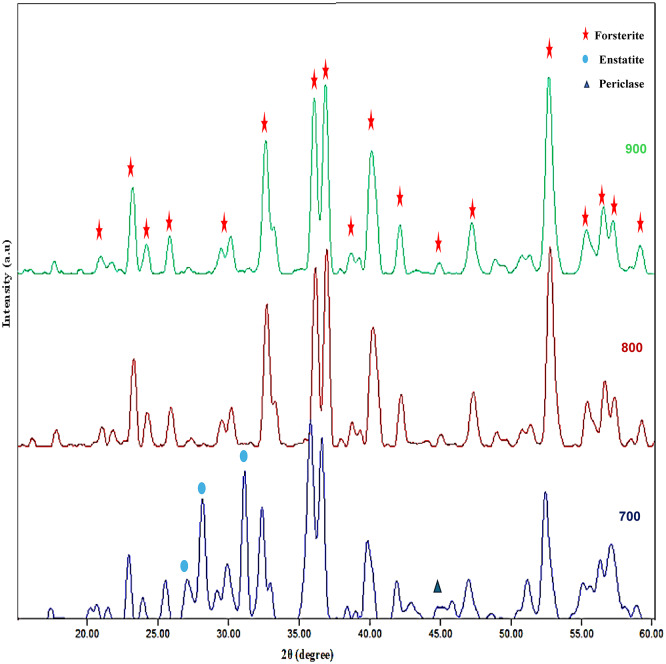
XRD pattern of nano forsterite at different temperatures from 700 to 900 °C for 2 h.

The X-ray diffraction (XRD) pattern of forsterite is shown in Fig. 1. Forsterite was prepared at different temperatures of 700, 800, and 900 °C as shown in Fig. 1. The XRD patterns of the samples revealed that the major peaks were observed at two thetas 17.56°, 20.77°, 23.04°, 24.02°, 25.66°, 29.95°, 32.47°, 35.91°, 38.53°, 40.00°, 47.09°, 50.58°, 51.13°, 52.55°, 55.19°, 56.41°, 63.05°, 64.12°, 67.39°, 73.37°, and 76.84° which correlated to [020], [110], [021], [101], [111], [121], [130], [131], [041], [140], [042], [103], [151], [222], [241], [061], [321], [223], [170], [134], and [270] planes, respectively, which were corresponding to the forsterite. This data was compatible with standard Crystallography Open Database (COD) no. 9,000,267 and exhibited orthorhombic structure with P b n m (62) space group. Also, other peaks appeared at the sample calcinated at 700 °C which corresponds to periclase (MgO) and proto enstatite according to standards COD no. 9013253 and COD no.1545542, respectively. So, it was clear that the pure forsterite phase was prepared at 800 °C and 900 °C but for the sample calcinated at 700 °C there were some other peaks of periclase (MgO) and proto-enstatite which relieved the incomplete solid-state reaction between periclase and silica at this temperature^20,21^.

**Fig. 2:**
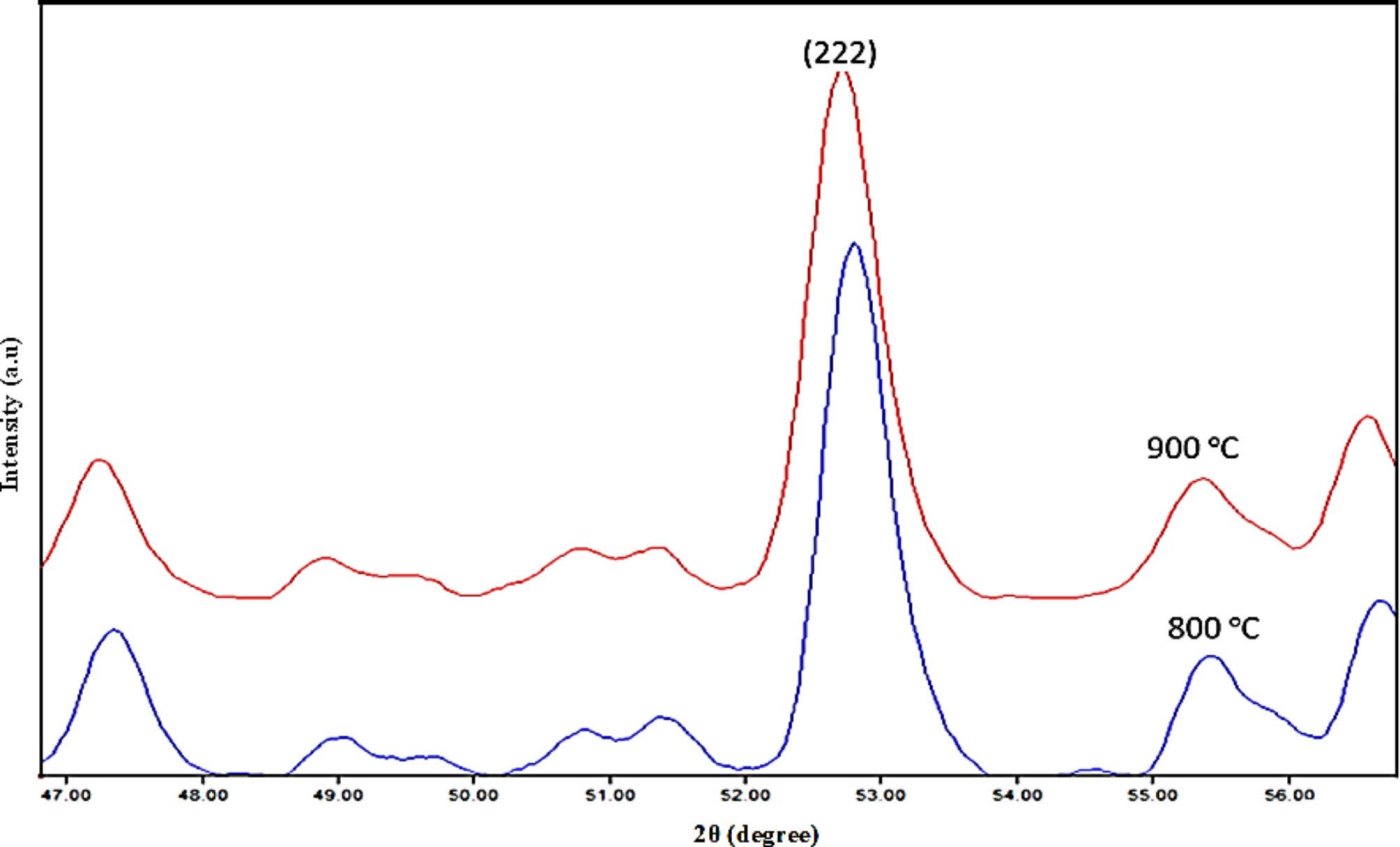
Zoom given the most intense peak (222).

Furthermore, as can be shown in Fig. 2 from the intense peak (222) at 2θ = 52.55°, by increasing the temperature of firing, the peak positions migrated to lower angles. This migration process means an increase in crystal size by increasing the firing temperature which is relieved by calculations of crystallite size through the Scherrer equation, where the average crystallite size at sample calcinated at 800 °C = 16.81 nm and 900 °C = 20.46 nm^22–24^. So, the sample that calcinated at 800 °C which gave a pure forsterite phase, and a small crystallite size was fully characterized by the other techniques and then applied as an adsorbent for EBD removal.

#### Transmission electron microscope (TEM)

**Fig. 3:**
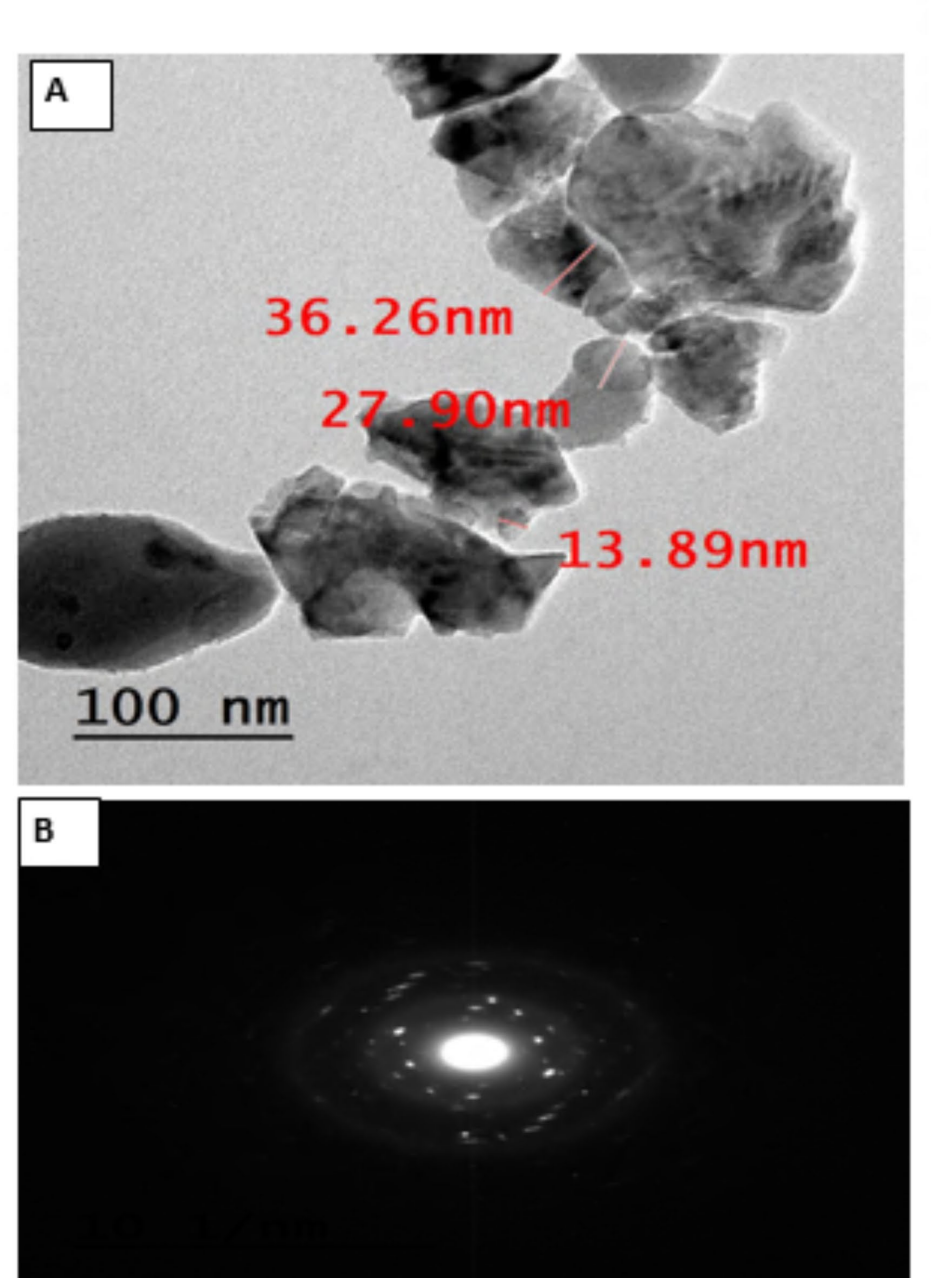
HR-TEM (A) and SAED (B) of the synthesized Forsterite nanoparticles.

As shown in Fig. 3(A), the synthesized nano forsterite underwent analysis utilizing TEM imaging to ascertain its shape and particle sizes. The TEM analysis revealed that Forsterite nanoparticles have a homogeneous matrix, orthorhombic, and highly crystalline nature with the average size of particles in the range from 13.89 to 36.26 nm with an average particle size was 26.2 nm which agreed with the average crystallite size calculated from XRD analysis which was 16.81 nm. The high-resolution TEM image (HR-TEM) of nano Forsterite, which is shown in selected area electron diffraction (SAED), clearly displays a well-organized lattice edge arrangement, indicating the material’s high degree of crystallinity (Fig. 3B)^2^.

#### Atomic force microscope (AFM)

**Fig. 4:**
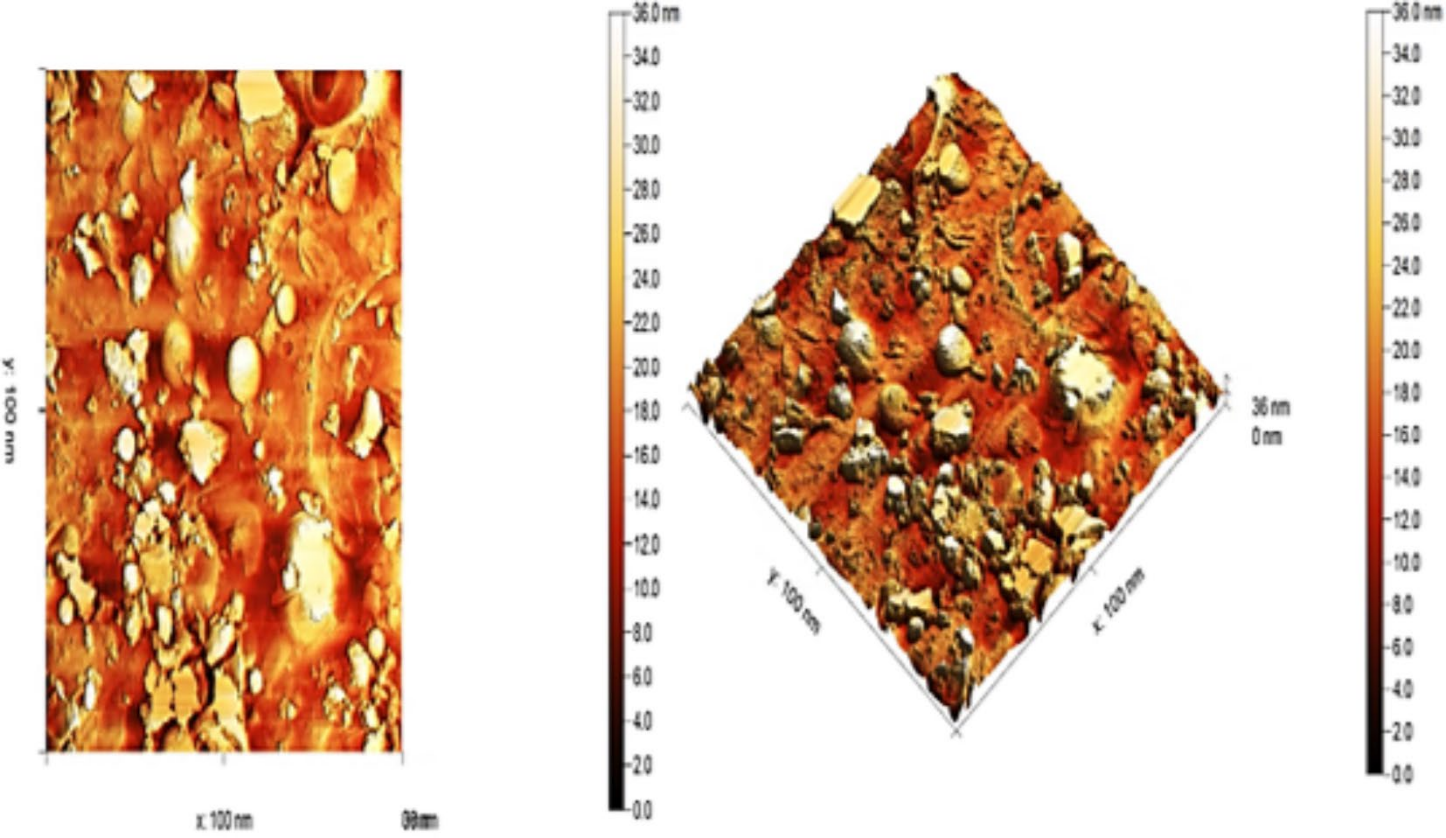
2D and 3D AFM images for Forsterite.

The surface topography and roughness of the Forsterite sample were analyzed using an atomic force microscope. Figure 4 showed 2D and 3D AFM images, respectively, for the forsterite nanoparticle. This figure showed an irregular surface with nonuniform shape grains. The AFM analysis showed that the grain size was 36 nm which is confirmed with XRD (average crystallite size = 16.81 nm) and TEM (average particle size = 26.2 nm) results with maximum peak height of 17.16 nm. Furthermore, the roughness of the samples was assessed by analyzing the average roughness (Ra = 4.11 nm) and root mean square of the roughness (Rq = 5.28 nm) from AFM images.

#### Brunauer-Emmett-Teller (BET)

The nitrogen adsorption-desorption isotherm was used to ascertain the surface area, pore volume, and characteristic pore size of the synthesized forsterite nanoparticles. Supplementary Figure (S1) indicated a type IV-(a)-H3 hysteresis loop isotherm of nano forsterite^25^. The largest amount of adsorbed volume was at P/P^0^ = 0.9. The surface area, the total pore volume, and the average pore size according to BET analysis were 65.77 m^2^/g, 0.137 cm^3^/g, and 4.19 nm, respectively, which indicated that nano forsterite has high surface area and mesopores structure^26^.

#### Contact angle study

By using a goniometer apparatus, the contact angle measurements have been carried out to assess the lipophilicity of the nano forsterite. As shown in Supplementary Figure (S2), the sample displayed a contact angle of 112.18$\:^\circ\:$ which is significantly larger than 90° and indicated a hydrophobic nature of the nano forsterite^27^.

After analyses of forsterite nanoparticles and comparing them to other previous nanomaterials that have similar properties such as mesoporous structure^28^, hydrophobicity^29^, and high surface area^30^, it was revealed that the nano forsterite can be used as an adsorbent in removing dyes such as EBD.

### Studying the factors that affect the adsorption process

Factors impacting dye removal from aqueous solutions, such as pH, adsorbent dose, initial dye concentration, stirring rate, and contact time, have been researched to determine the optimal parameters exhibiting the maximum removal effectiveness of the EBD dyes under study.

#### Effect of pH

**Fig. 5:**
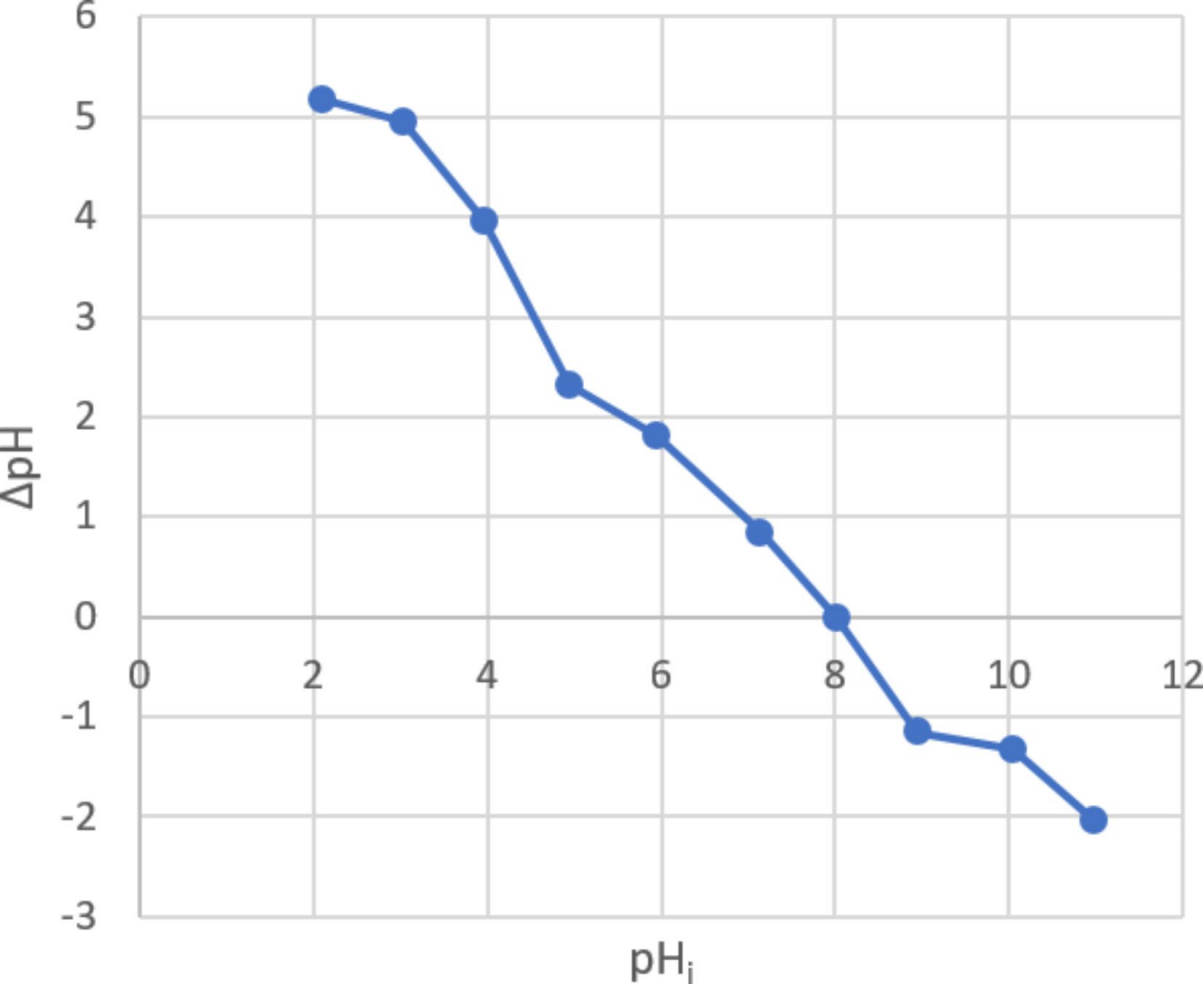
Zero-point charge measurement of forsterite nanoparticles.

**Fig. 6:**
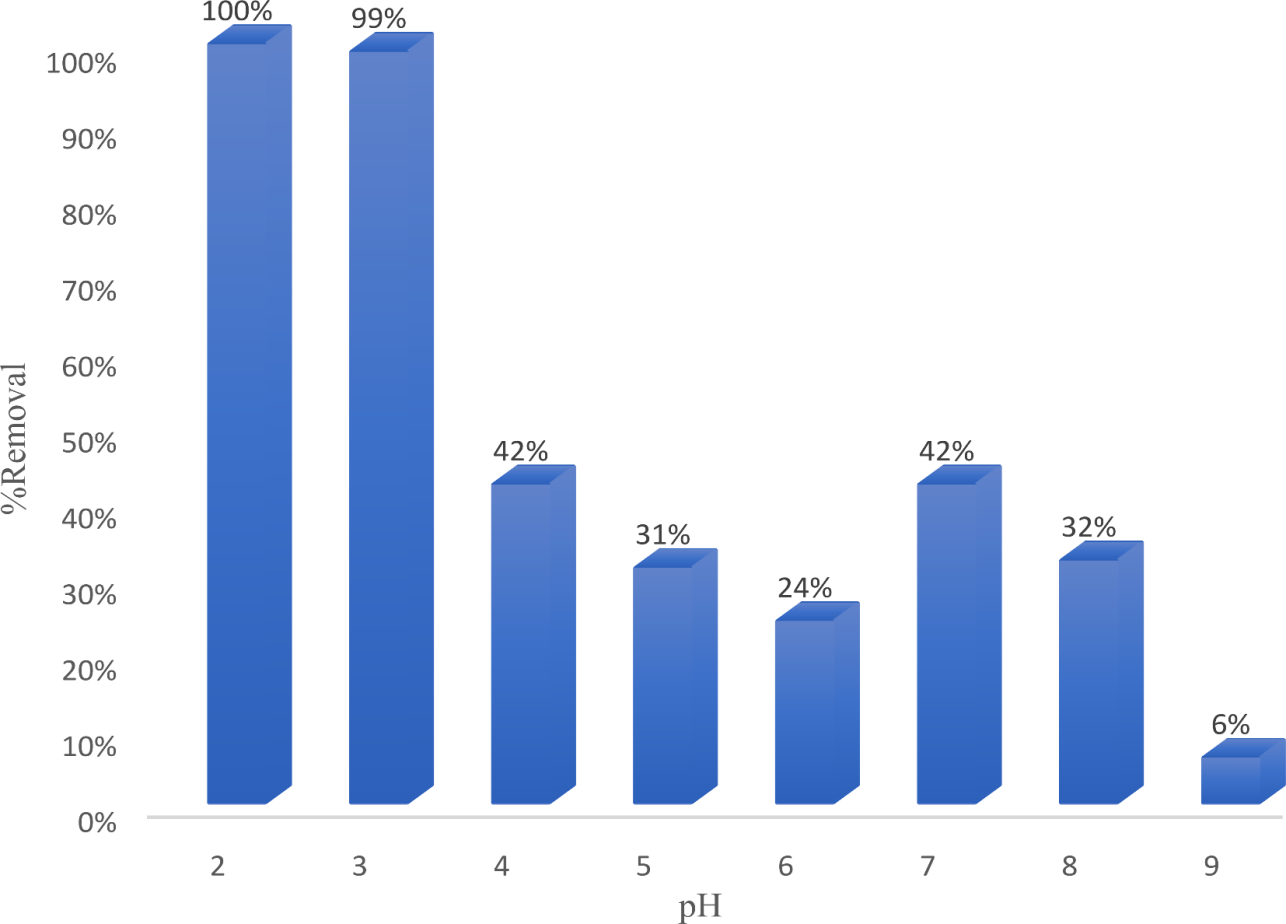
Effect of pH on the removal of EBD by using 0.1 g Nps/100 mL, 10 ppm dye concentration, time 10 min, and acceleration speed.

The pH of the solution is a crucial determinant of the adsorption process as it influences the adsorption capacity. The effect of pH on adsorption varies depending on the nature of the organic dye (whether it is anionic or cationic) and the interaction of the forsterite nanoparticles^31,32^. The adsorption process is more effective in acidic environments because the pH at which the surface charge of forsterite becomes zero (pH_pzc_) is 8, as seen in Fig. 5. Below pH_pzc_, the surface charge is positive, and above it, it becomes negative^33^. Consequently, there is a strong electrostatic attraction between the nano forsterite and the anionic dye, facilitating their interaction. As depicted in Fig. 6, when using 10 ppm of anionic dye at pH 2 and 3, the removal percentage was nearly 100%. However, it gradually decreased and reached 6% at pH 9 due to the deprotonation of the sulphonate group. This resulted in the dye acquiring a negative net charge, which was counteracted by the anionic forsterite nanoparticle^32^.

####  Effect of adsorbent dosage

**Fig. 7:**
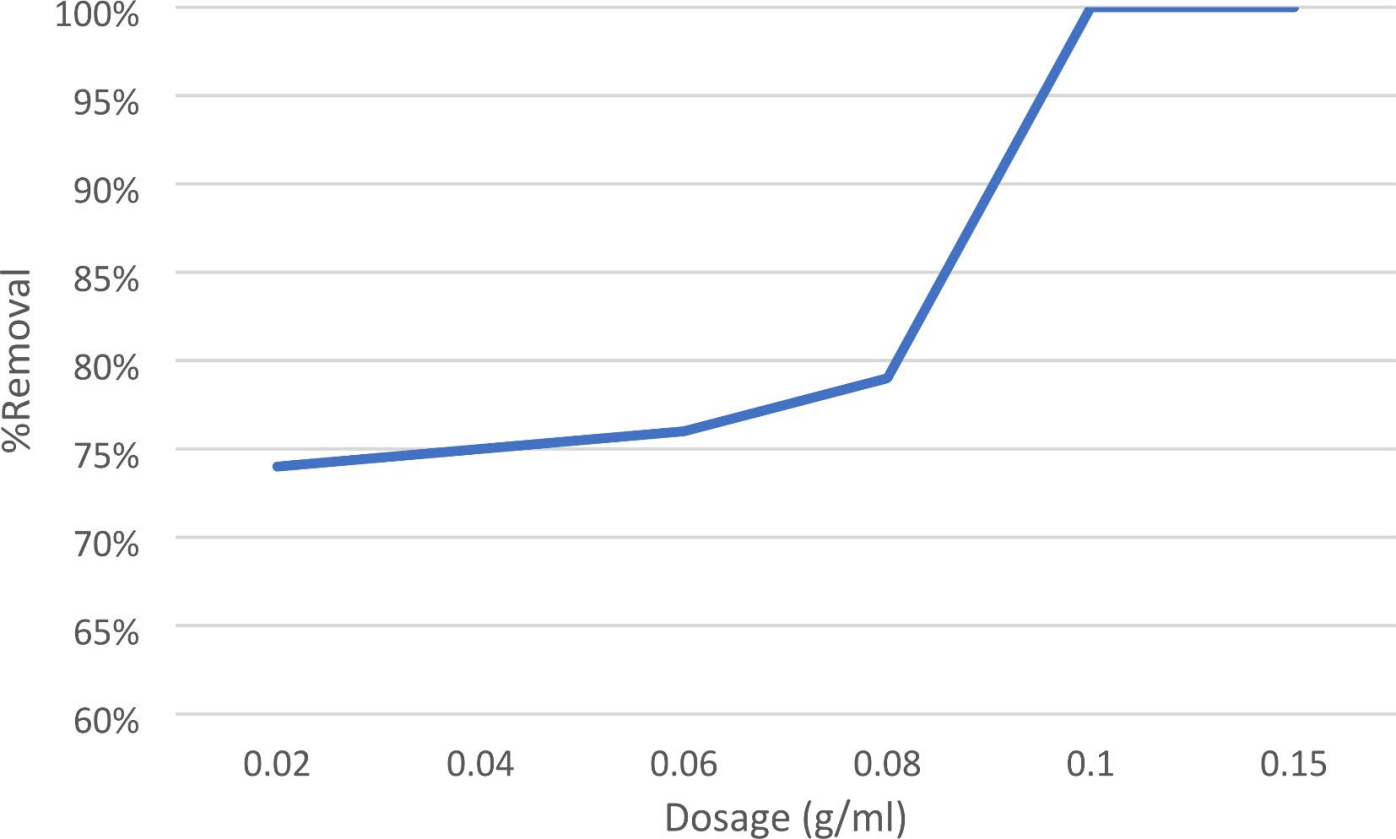
Adsorbent dosage effect on removal of EBD at pH 3, 10 ppm dye concentration, time 10 min, and acceleration speed.

The impact of adsorbent dose was examined by altering the quantity of nano forsterite from 0.02 to 0.15 g/100 mL while maintaining an initial concentration of 10 mg/L at pH 3. This is shown in Fig. 7. The elimination percentage rose from 76 to 100% by increasing the dose of absorbent from 0.02 to 0.1 g. The reason for this is that raising the dosage of the adsorbent improves the removal percentage by increasing the number of active sorption sites on the surface of the adsorbent^34^.

#### Effect of initial dye concentration

**Fig. 8:**
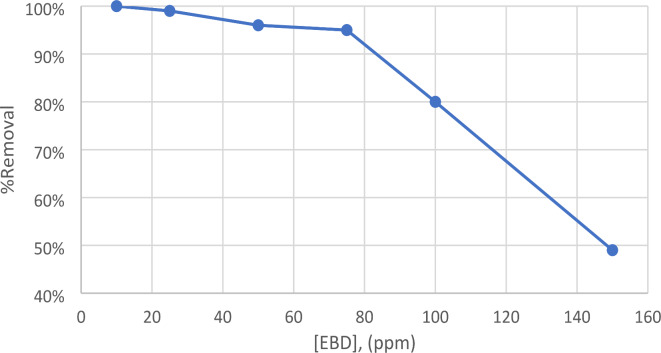
Effect of initial dye concentration on the removal of EBD at pH 3, 0.1 g Nps/100 mL, time 10 min, and acceleration speed.

Dye adsorption is a phenomenon where dye molecules accumulate at the boundary between a solid and a liquid, including the transfer of mass. The study focused on examining the influence of the initial dye concentration while keeping the other parameters constant. According to Fig. 8, the removal percentage reduces from 95 to 80% when the first dye concentration rises from 80 to 100 ppm. Similarly, the removal percentage decreases from 80 to 49% as the initial dye concentration increases from 100 to 150 ppm. The higher initial dye concentration resulted in a decrease in the removal % due to a reduction in the number of active sites on the adsorbent. This is especially apparent when the original concentration of EBD is more than 100 ppm. One additional aspect that causes the removal % to decline is the barrier to mass transfer between the liquid phases (which include the dye, water, and other interfering substances) and the solid phase (the forsterite adsorbent). Nevertheless, the EBD served as a crucial impetus to surmount this hindrance in the transfer of mass. As a result, the barrier to mass transfer decreased, leading to an improvement in the efficiency of dye removal as the initial concentration decreased. Similar results have been seen in prior research^35–38^.

#### Effect of contact time

**Fig. 9:**
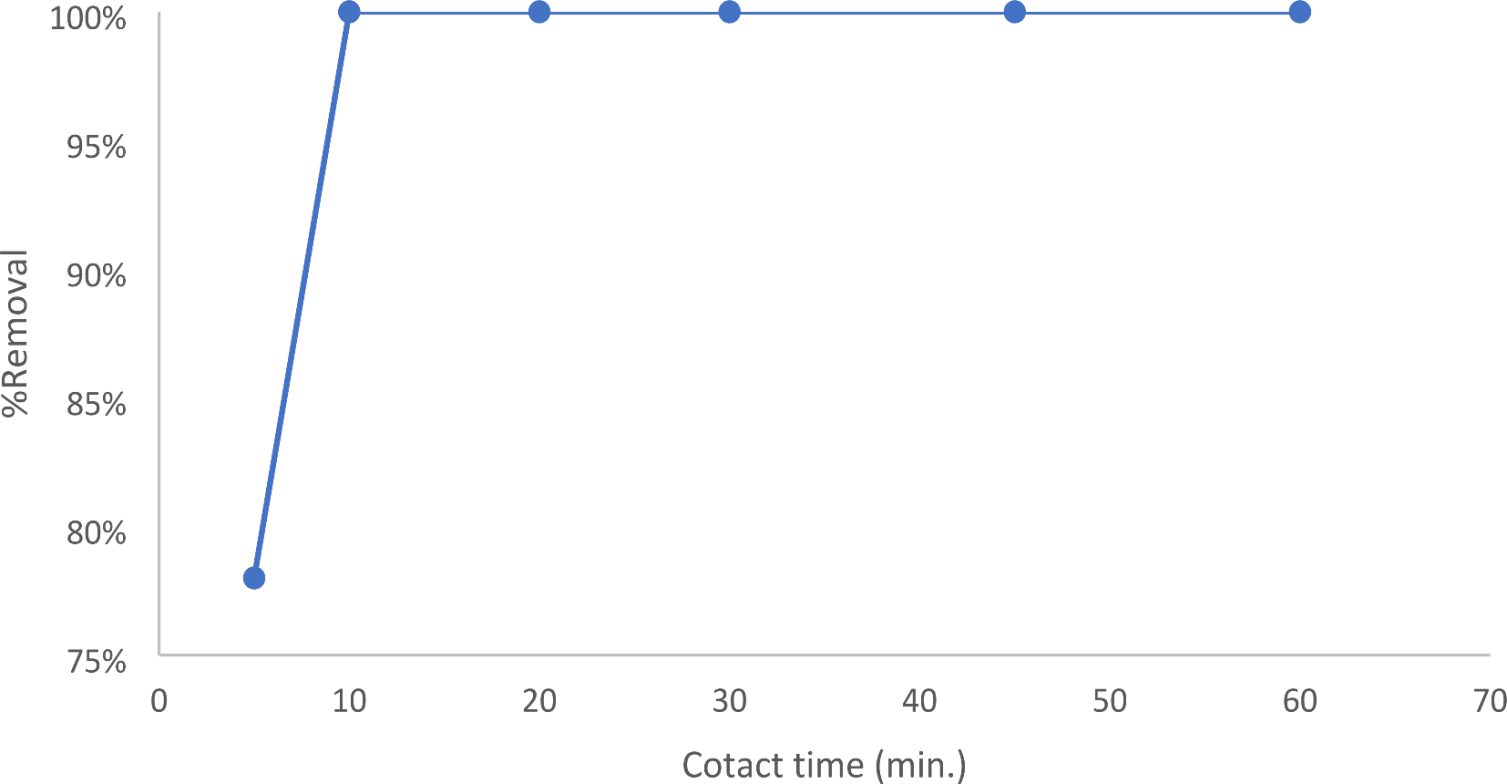
Contact time factor on the removal of EBD at pH 3 using 0.1 g Nps/100 mL, 10 ppm dye concentration, and acceleration speed.

The equilibrium time is a crucial factor in an efficient wastewater treatment system. Adsorption kinetics is a crucial parameter for comprehending the process of adsorption and evaluating the effectiveness of the adsorbent. For a material to be successful as an adsorbent, it must have a quick and quantifiable adsorption rate. An investigation was conducted to examine the influence of contact duration on the adsorption of dye, with time intervals ranging from 5 to 60 min. Figure 9 illustrates that the clearance percentage is almost 100% at 10 min and remains constant at a pH of 3 and a dye concentration of 10 ppm.

#### Acceleration speed’s effect

**Fig. 10:**
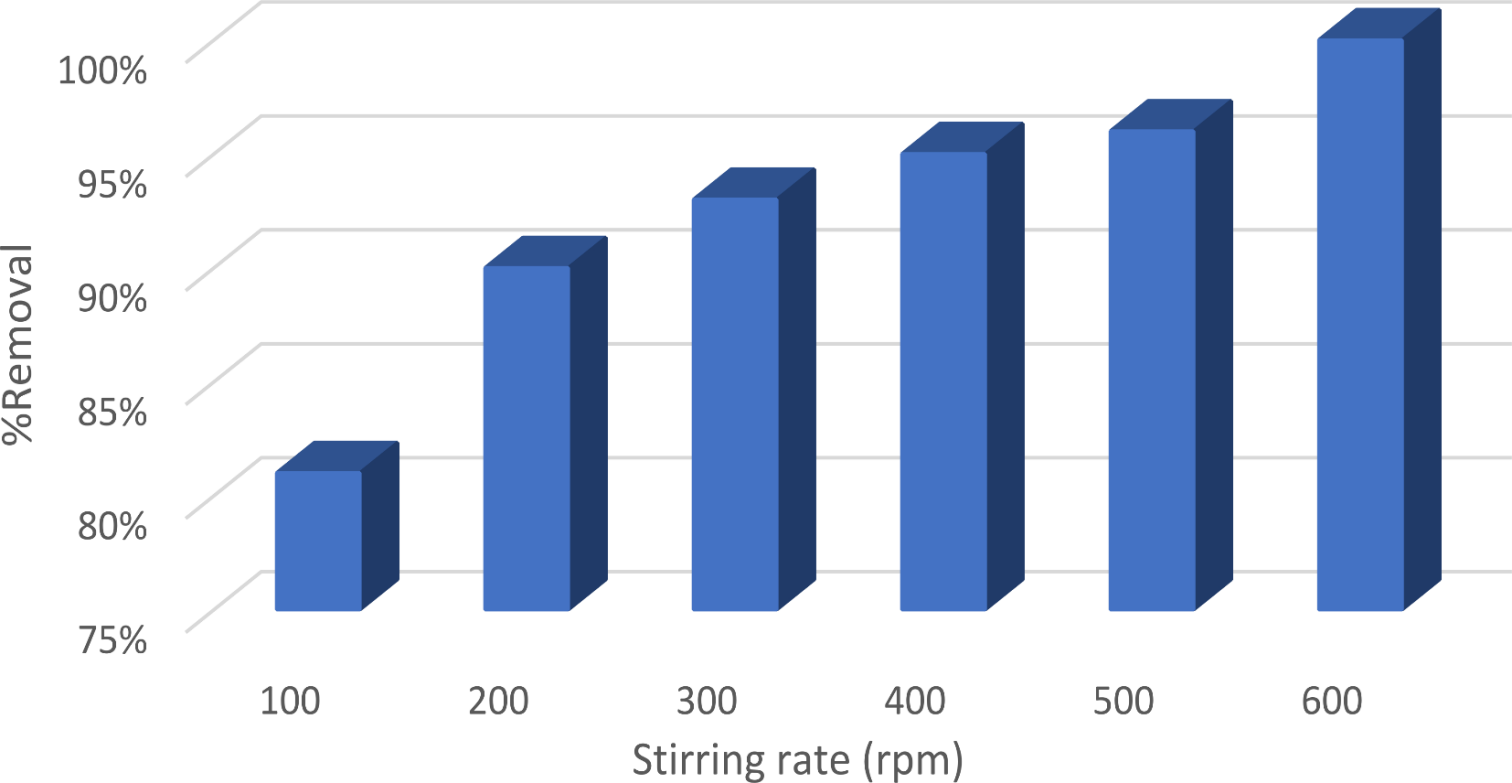
Effect of agitation speed on the removal of EBD at pH 3 using 0.1 g Nps/100 mL, 10 ppm dye concentration, and time 10 min.

The impact of agitation speed is seen in Fig. 10. The agitation speed is increased from 100 to 600 rpm, resulting in a shift in the removal percentage from 81 to 100%. This may be attributed to the fact that agitation decreases the resistance of the boundary layer while simultaneously enhancing the mobility of the system. Moreover, increasing the agitation speed decreases the impact of external mass transfer, hence promoting close interaction between the two phases (adsorbent and adsorbate)^39^.

### Adsorption isotherm

Adsorption isotherms were conducted utilizing Freundlich, Langmuir, and Temkin equations for water and wastewater treatment applications^40^.

####  Langmuir isotherm

This model proposes that adsorption occurs at certain uniform sites inside the adsorbent, without any substantial interaction between the adsorbed species. The adsorbent reaches saturation when a monolayer of molecules is adsorbed onto its surface^40^. This model has the form (Eq. 1):

1 $${q_e}=\left( {{q_{\hbox{max} }}b{C_e}} \right)/\left( {1+b{C_e}} \right)$$

The linear form is the following (Eq. 2):

2 $${C_e}/{q_e}=\left( {1/{q_{\hbox{max} }}} \right){C_e}+1/\left( {{q_{\hbox{max} }}b} \right)$$

The variable q_e_ represents the mass of adsorbate per gram of sorbent. q_max_, measured in milligrams per gram, represents the maximum adsorption capacity. Ce, measured in milligrams per liter, represents the concentration of the equilibrium solution. Lastly, b, measured in liters per milligram, represents the coefficient of affinity, which is connected to the energy of adsorption.

**Fig. 11:**
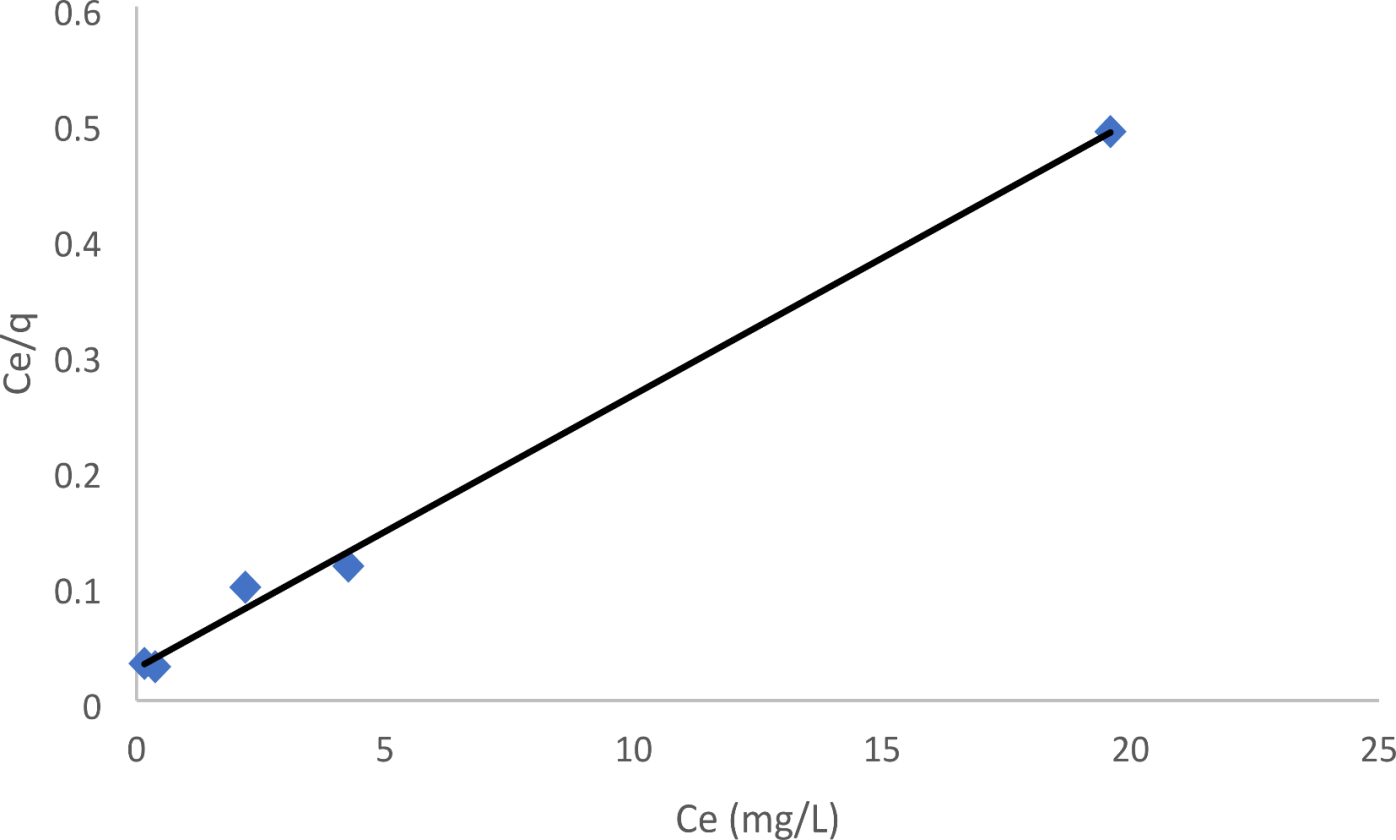
Langmuir adsorption isotherm of EBD onto forsterite Nps.

The values of q_max_ and b were obtained by analyzing the linear relationship between C_e_/q_e_ and C_e_, as depicted in Table 2; Fig. 11. The maximum adsorption capacity of EBD per gram of nanoparticle forsterite was found to be 42.30 mg, while the Langmuir constant equilibrium, b, was determined to be 0.85. The R^2^ value of 0.996 indicated a strong match between the sorption data and the Langmuir Isotherm model. To verify the fundamental properties of the Langmuir isotherm, the separation factor (R_L_) was computed for the highest concentration of adsorbate (C_o_) in units of milligrams per liter (mg/L) using Eq. (3).

3 $${{\text{R}}_{\text{L}}}={\text{ 1}}/{\text{ }}\left( {{\text{1}}\,+\,{{\text{C}}_{\text{o}}}{\text{b}}} \right)$$

The R_L_ value indicates the degree of favorability of the adsorption isotherm. Adsorption is categorized as irreversible if R_L_ = 0, favorable if 0 < R_L_ < 1, linear if R_L_ = 1, and unfavorable if R_L_ > 1^41^. Table 1 demonstrated that the R_L_ values imply good equilibrium sorption for concentrations ranging from 10 to 99 mg/L.

**Table 1:** Values of separation factor (R_L_).

C_0_ (mg/L)	*R*_L_
10	0.10
26	0.04
47	0.02
78	0.01
99	0.01

####  Freundlich isotherm

This model is predicated on the premise that the sorption system is comprised of several non-uniform layers and surfaces, including restricted sorption sites and potential energy interactions^40^. The Freundlich model is expressed mathematically, as seen in Eq. (4).

4 $${q_e}={K_f}C{e^{1/n}}$$

To linearize Eq. (4), use the following constants and logarithms:

5 $$log{q_e}=log{K_f}+(1/n)logCe$$

Where the Freundlich constants are K_f_ and n, n indicates favorability, and K_f_ is the adsorbent’s capacity. Values of 1/n below 1 imply strong adsorption at low concentrations, whereas the increase of adsorption with concentration becomes less significant at higher concentrations, and vice versa^42^. Higher K_f_ values result in increased adsorption intensity^43^.

**Fig. 12:**
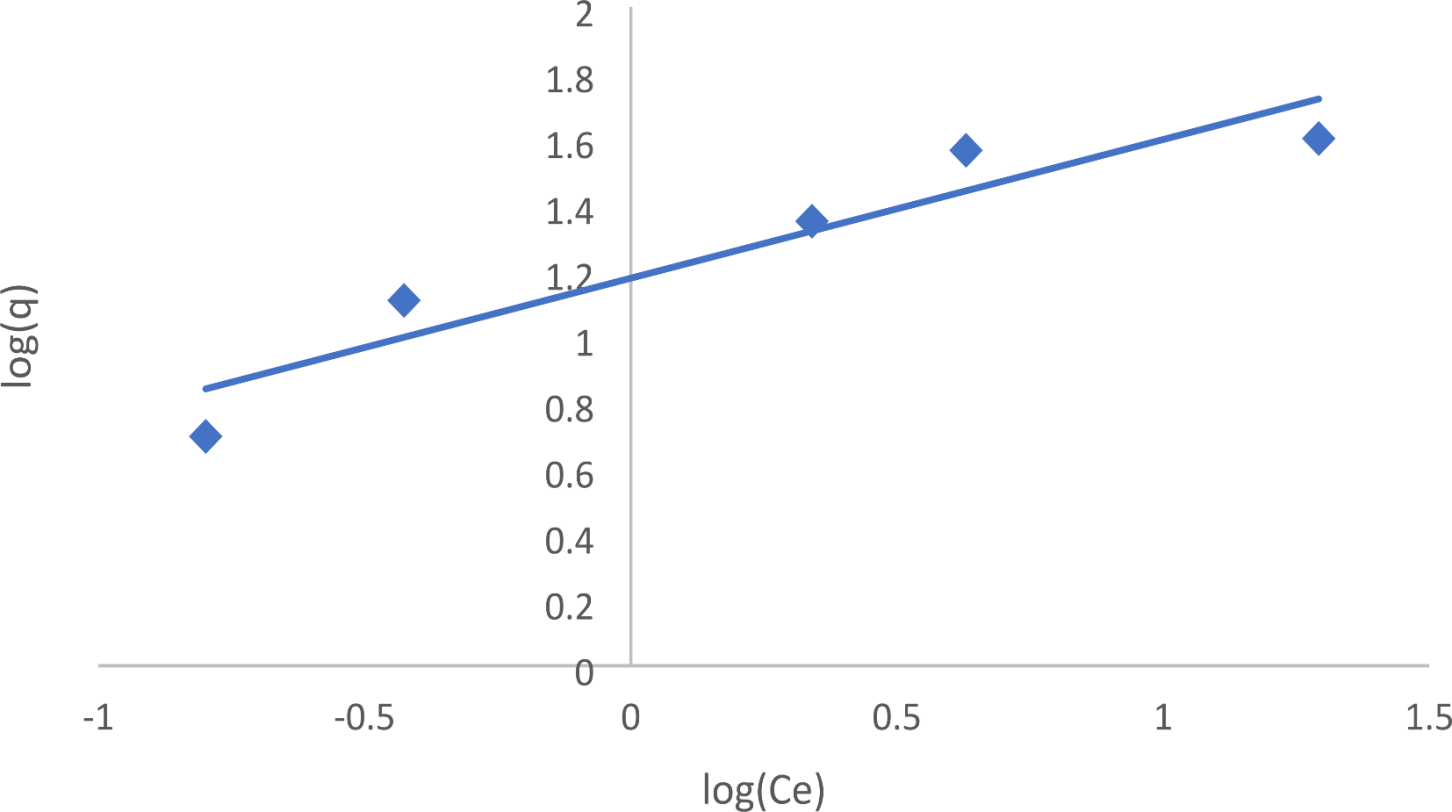
Freundlich adsorption isotherm of EBD onto forsterite Nps.

As shown in Table 2; Fig. 12, the Freundlich isotherm showed that the 1/n values are between 0 and 1, which indicated that EBD adsorption onto forsterite is favorable, and the surface of forsterite Nps with the dye is strongly bound, where values of n in the 1–10 range indicated that this dye favors adsorption onto forsterite Nps.

**Table 2:** Langmuir, Freundlich, TEMKIN and R^2^ values of EBD adsorption on nano forsterite.

Isotherm	Results
Langmuir isotherm	q_max_ = 42.3 mg/g b = 0.85 L/mg R^2^ = 0.996
Freundlich isotherm	K_f_ = 15.01 (mg/g) (L/g)^1/*n*^ *N* = 2.38 1/*n* = 0.42 R^2^ = 0.886
Temkin isotherm	B = 7.57 J/mol b = 0.327 A = 13.55 L/mg R^2^ = 0.940

#### Temkin isotherm

Two presumptions underlie this isotherm. Firstly, it assumes that the adsorption heat of the whole layer of molecules drops linearly as the coverage rises, which is due to the interactions between the absorbent and adsorbate. Moreover, a consistent distribution of binding energies that approaches the maximal binding energy characterizes the adsorption^40^. The Temkin isotherm is widely used for non-uniform sorption heat distribution^44^.

6 $${q_e}=(RT/b)lnA+(RT/b)ln{C_e}$$

**Fig. 13:**
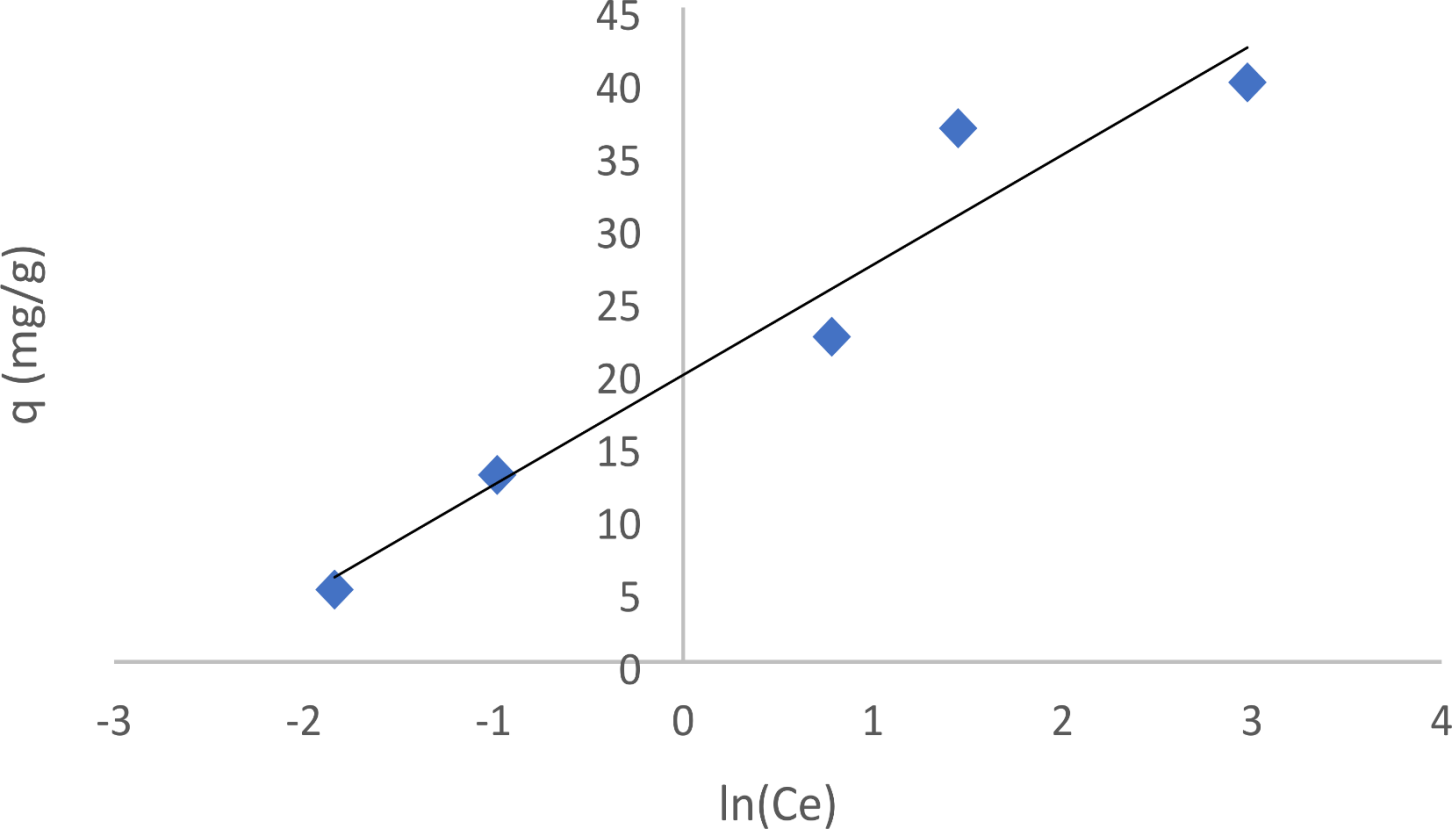
Temkin adsorption isotherm of EBD onto forsterite Nps.

The equilibrium binding constant, A (L/mol), reflects the maximum binding energy. B is the adsorption’s heat constant where B = *RT*/*b* constant associated with the heat of sorption (J/mol) calculated from the Temkin plot (*q*_*e*_ against ln C_*e*_); R = universal gas constant (8.314 J/mol.K) T = temperature (298 K) ^45^. As shown in Fig. 13, the Temkin isotherm was obtained straight line. Temkin isotherm constant (b) can be obtained from the slope, B = RT/b, and the Temkin isotherm equilibrium binding constant (A) can be obtained from the intercept, (RT/b)lnA, as indicated in Table 2.

According to the R^2^ results, it is more appropriate to use the Langmuir isotherm for EBD adsorption on forsterite Nps, with a correlation coefficient (R^2^) of 0.996 demonstrating that the Langmuir adsorption model accurately simulates the experimental results and that the adsorption occurred as a monolayer^7^. This means that the adsorbent’s surface, the arrangement of molecules on the surface, and the type of interaction between EBD and the adsorbent are all factors in the sorption capabilities.

### Adsorption kinetics

This work assessed the kinetics of EBD removal by forsterite Nps utilizing both pseudo-first and pseudo-second order kinetics.

Equation (7) states that the pseudo-first-order module represents the quantity of adsorbate that has been adsorbed over time (t) on the adsorbent surface. When one or more concentrations significantly influence the rate of the reaction, it is consistent with the reaction. It mainly defines the adsorption on heterogeneous adsorbent surfaces.

7 $$\log ({\text{q}}_{{\text{e}}} - {\text{q}}_{{\text{t}}} ) = \log {\text{q}}_{{\text{e}}} - ({\text{k}}_{1} /2.303){\text{t}}$$

However, the pseudo-second-order module was designed to pretend the real reaction as closely as possible. It believes that a chemisorption process is the slowest^46^ and can be expressed using the following Eq. (8).

8 $${\text{t}}/{\text{q}}_{{\text{t}}} = 1/{\text{k}}_{2} {\text{q}}_{{\text{e}}}^{{\text{2}}} + (1/{\text{q}}_{{\text{e}}} ){\text{t}}$$

where k_1_ and k_2_ are the first-order and second-order rate constants, respectively, and q_e_ and q_t_ are the quantities of dyes adsorbed on the surface of the forsterite Nps at equilibrium and at the time (t), respectively, in mg/g.

**Fig. 14:**
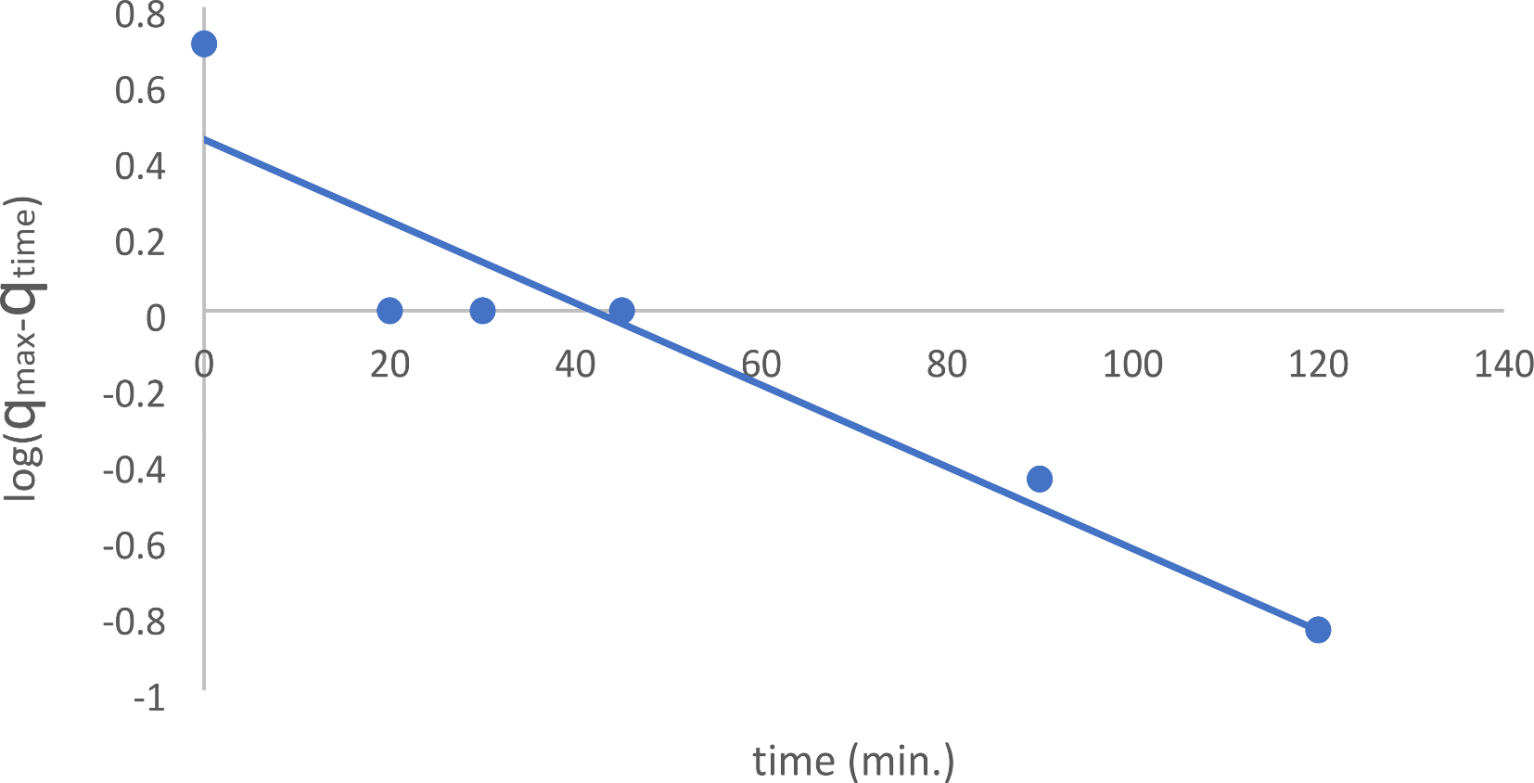
Pseudo-first order kinetics of the removal of EBD.

**Fig. 15:**
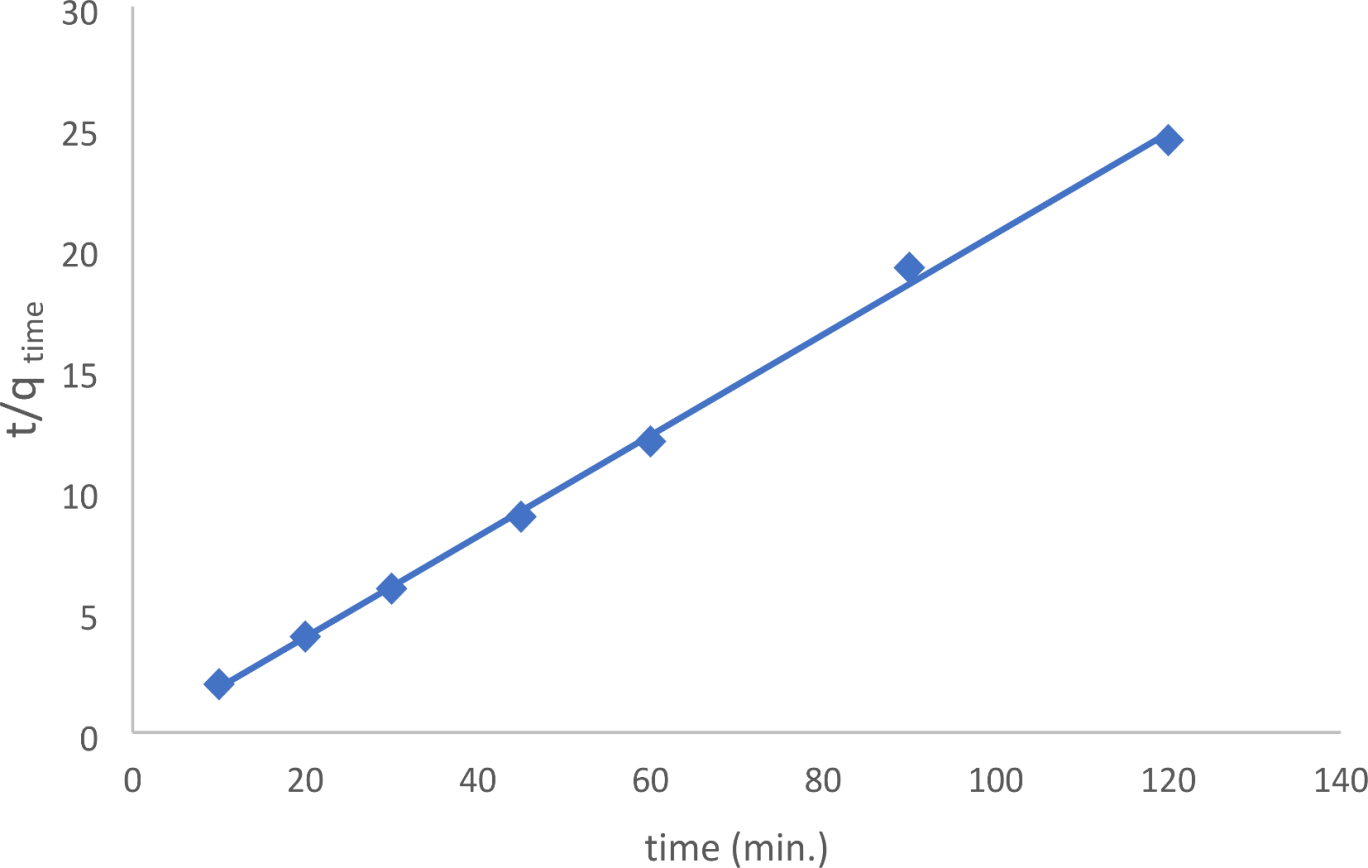
Pseudo-second order kinetics of the removal of EBD.

Table 3 listed and determined each of the previously mentioned parameters. As shown in Fig. 14, low correlation values are seen in the pseudo-first-order kinetic model (R^2^ = 0.894). Furthermore, between the actual and theoretical findings, there was a discernible discrepancy in the equilibrium adsorption capacity (q_e_), suggesting that the pseudo-first-order fit to the experimental data was not very good. Conversely, the outcomes of pseudo-second-order kinetics exposed that a linear fit was achieved with remarkably extraordinary correlation coefficients (R^2^ = 0.998), as shown in Fig. 15. In the case of pseudo-second-order kinetics, the theoretical q_e_ values also fairly agree with the experimental results.

**Table 3:** The kinetic parameters for EBD adsorption on forsterite nps.

Kinetic model	Parameters	Results
Pseudo-first order	q_e_(exp.) mg/g	5.05
	q_e_(theo.) mg/g	2.82
	K_1_ (min^-1^)	0.02
	R^2^	0.894
Pseudo-second order	q_e_(exp.) mg/g	5.05
	q_e_(theo.) mg/g	4.8
	K_2_ (min^-1^)	– 0.188
	R^2^	0.998

### Regeneration

**Fig. 16:**
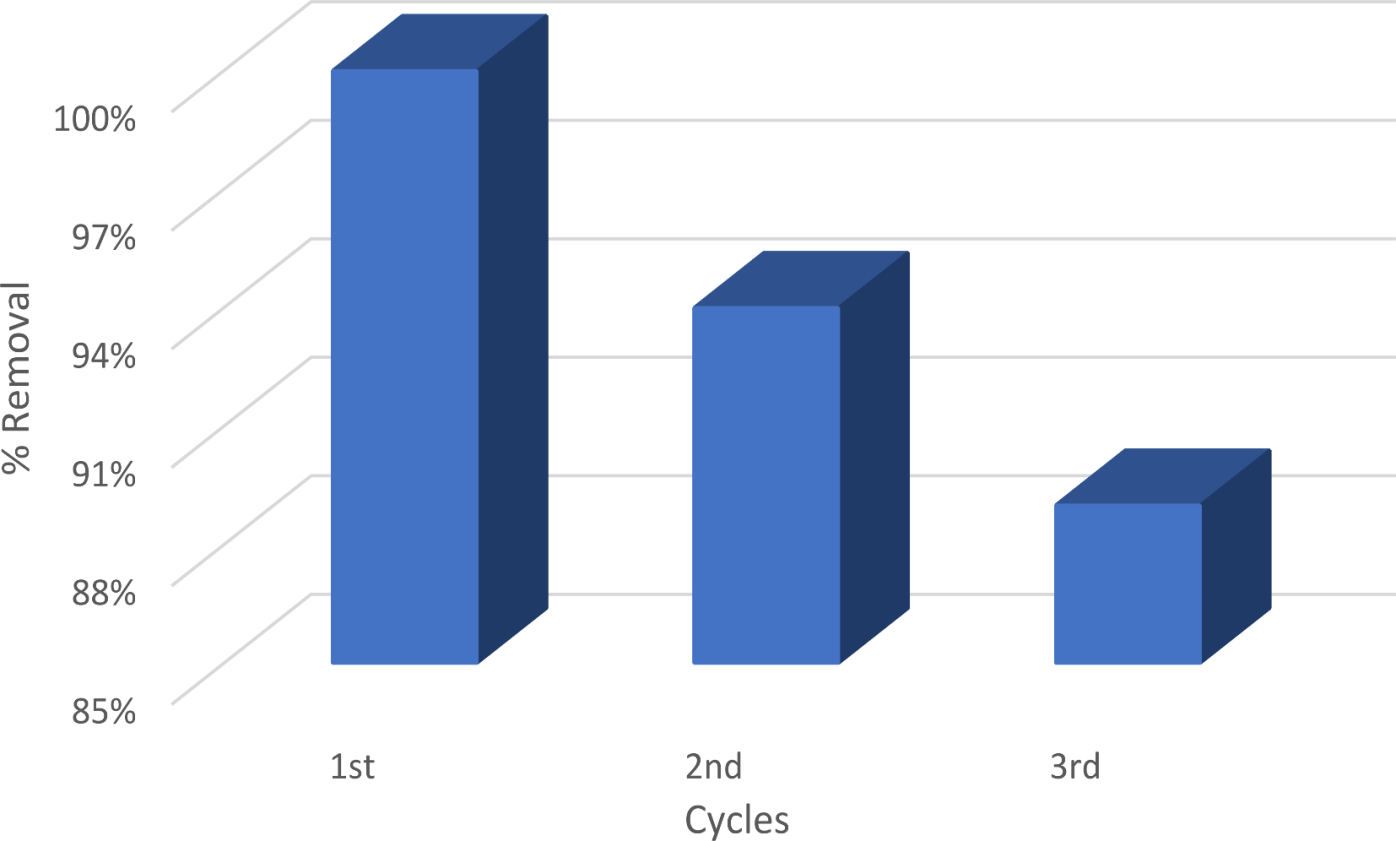
Number of cycles of regeneration.

To validate the environmental friendliness and cost-effectiveness of forsterite, it is crucial to carry out the regeneration process. To carry out the desorption phase, the adsorbent is retrieved after the adsorption procedure and subjected to washing with a solution of 0.01 mol/L of NaOH and HCl, followed by rinsing with distilled water. Forsterite nanoparticles (NPs) were used for three cycles, after that the number of active sites decreased, exhibiting a removal efficacy of around 90%, as seen in Fig. 16. The findings demonstrated that forsterite was a very economical material.

### Comparison between different types of adsorbents

According to the data presented in Table 4, it can be deduced that forsterite nanoparticles demonstrate faster and more effective elimination of EBD compared to other materials investigated. The nano forsterite exhibited a faster removal of the EBD after 10 min compared to magnetic spinel ZnFe_2_O_4_ nanomaterial, layered double hydroxides (LDH), and durian husk. Furthermore, it was found to be effective at a lower recommended dosage than durian husk material. The observed outcome can be ascribed to the distinctive characteristics of these nanoparticles, as elucidated in the referenced studies.

**Table 4:** Comparison between different types of adsorbents for removal of EBD.

Adsorbent	Treatment	Adsorption isotherm	Contact time	Removal %	Dosage g/l	Initial conc. ppm	Refs.
Magnetic spinel ZnFe_2_O_4_ nanomaterial	Adsorption	Freundlich	60 min.	95%	0.5	50	^33^
Layered double hydroxides (LDH)	Adsorption	Langmuir	60 min.	99%	1	90	^47^
Durian husk	Adsorption	Sips	300 min.	95.95%	10	75	^48^
Forsterite	Adsorption	Langmuir	10 min.	100%	1	80	This work

## Experimental

### Chemicals and materials

All materials and apparatus were supported in the supplementary materials.

### Preparation of forsterite NPs

The sol-gel method was used to prepare Forsterite. Magnesium chloride hexahydrate and tetraethyl ortho silicate (TEOS) were used as starting magnesium and silicon precursors. TEOS was hydrolyzed with a mixed mixture of 125 mL of distilled water and 25 mL of ethanol (EtOH) and stirred for 1 h at 80 °C. Following that, 1 molar of MgCl_2_ was added to the solution and stirred for 30 min under the same conditions. The pH of the solution was adjusted to 10 by adding NH_4_OH. After that, the solution was left for 24 h and allowed to form a highly viscous solution. The highly viscous solution was filtered, and the precipitate was dried and then calcined at 700 °C, 800 °C and 900 °C for determining the optimum temperature which give pure Forsterite  ^49^.

### Characterization of nanoparticles

The crystallinity of the material was measured using a (D8 Discover, Bruker diffractometer - Germany). Measurements were performed at 40 kV and 40 mA current within 3° ≤ 2θ ≤  80º. The surface topography and roughness of the Forsterite sample were analyzed by using an atomic force microscope (AFM). A transmission electron microscope (TEM) was performed using (a JEOL-jem 2100, Japan) to analyze the internal characteristics. The specific surface area was determined after degassing Forsterite nanoparticles, for 10 h. by a Quanta chrome automated gas sorption system using nitrogen as the adsorbate. The contact angle is measured by using a goniometer apparatus.

### Adsorption studies

Adsorption studies were carried out at a constant temperature of (25.0 ± 1.0 °C) using 0.1 g of adsorbent and 100 mL of dye solution about 1 g/L. 0.1 M of NaOH or HCl solutions were used to adjust the pH of the tested solutions. The studies were performed by shaking the mixture solution at a constant rate in a 250 mL conical flask. The adsorbent was separated from all samples using a 45 μm polyethylene membrane filter after the predetermined time, and the analysis was conducted three times. A UV-Vis spectrophotometer (UVmini-1240, Shimadzu) was used to measure the concentration of EBD in the samples collected at certain time intervals at a wavelength of 620 nm.

Many variables were investigated such as the pH value, adsorbent dosage, initial dye concentration, shaking speed, and contact time. The following Eq. (9) was used to calculate the % dye removal:

9 $$\% Removal=[(C0 - Ce)/C0] \times 100$$

Where C_0_ is the initial concentration of dye (mg/L) while C_e_ is the dye concentration after adsorption (mg/ L). Furthermore, the adsorption capacity of the adsorbent, q_e_ (mg dye per g dry adsorbent) can be calculated using the following Eq. (10):

10 $${{\text{q}}_{{\text{max}}}}={\text{ }}\left( {{{\text{C}}_{\text{o}}}-{{\text{C}}_{\text{e}}}} \right){\text{ }} \times \left( {{\text{v}}/{\text{w}}} \right)$$

where V (in liter) is the solution volume and w (in gram) is the amount of dry adsorbent.

## Conclusion

The sol-gel technique was used to synthesize forsterite nanoparticles, which were then analyzed using XRD, TEM, AFM, contact angle, BET, and zeta potential. At a pH of 3 and an initial dye concentration of 10 ppm, 0.1 g of forsterite nanoparticles were used as an adsorbent for EBD. Within 10 min and at ambient temperature, the percentage of elimination reached almost 100%. The experimental data exhibited a strong match with a Langmuir model, using the greatest regression coefficient of R^2^ = 0.996. The maximum adsorption capacity, q_max_, was measured to be 42.3 mg/g. The kinetics data suggested that the sorption process is controlled by a pseudo-second-order mechanism. Studies on regeneration demonstrated that forsterite nanoparticles can be employed for three cycles with superior removal efficiency. The properties of forsterite nanoparticles enhanced their potential in the domain of wastewater treatment for the elimination of EBD.

## Electronic supplementary material

Below is the link to the electronic supplementary material.

Supplementary Material 1

## Data Availability

All data generated or analyzed during this study are included in this published article [and its supplementary information files].
